# Sensor Fusion for Underwater Vehicle Navigation Compensating Misalignment Using Lie Theory

**DOI:** 10.3390/s24051653

**Published:** 2024-03-03

**Authors:** Da Bin Jeong, Nak Yong Ko

**Affiliations:** Department of Electronic Engineering, Interdisciplinary Program in IT-Bio Convergence Systems, Chosun University, Gwangju 61452, Republic of Korea; jjdabin@naver.com

**Keywords:** Lie theory, misalignment, attitude, navigation, Kalman filter, underwater vehicle

## Abstract

This paper presents a sensor fusion method for navigation of unmanned underwater vehicles. The method combines Lie theory into Kalman filter to estimate and compensate for the misalignment between the sensors: inertial navigation system and Doppler Velocity Log (DVL). In the process and measurement model equations, a 3-dimensional Euclidean group (SE(3)) and 3-sphere space ($S^{3}$) are used to express the pose (position and attitude) and misalignment, respectively. SE(3) contains position and attitude transformation matrices, and $S^{3}$ comprises unit quaternions. The increments in pose and misalignment are represented in the Lie algebra, which is a linear space. The use of Lie algebra facilitates the application of an extended Kalman filter (EKF). The previous EKF approach without Lie theory is based on the assumption that a non-differentiable space can be approximated as a differentiable space when the increments are sufficiently small. On the contrary, the proposed Lie theory approach enables exact differentiation in a differentiable space, thus enhances the accuracy of the navigation. Furthermore, the convergence and stability of the internal parameters, such as the Kalman gain and measurement innovation, are improved.

## 1. Introduction

Sensing and monitoring in an underwater environment rely heavily on the reliable navigation of an underwater vehicle. The underwater vehicles installed with sensors for environmental monitoring can navigate through sites of interest and accumulate the sensor data for analysis. The inertial navigation system (INS) and Doppler velocity log (DVL) play pivotal roles in ensuring accurate underwater navigation [1,2,3,4,5]. Misalignment between the INS and DVL results in a degradation of overall navigation performance. This misalignment leads to persistent challenges in achieving successful navigation.

This paper proposes a method that estimates and compensates for the misalignment between INS and DVL in underwater vehicles. The proposed method incorporates Lie theory into Kalman filter (KF) to estimate explicitly the misalignment as well as the navigation variables such as the position and attitude for which the misalignment is compensated. Arithmetic operations based on the assumption that misalignment variables constitute linear space require approximations and cause instability in estimation. This limitation calls for Lie theory for rigorous implementation of the estimation. The use of Lie theory makes the estimation more accurate and stable.

INS and DVL are generally used as sensors for the navigation of underwater vehicles. In [1], for robust INS/DVL navigation, the complexity of the underwater environment is evaluated, and the navigation filters are adaptively employed depending on the complexity [1]. A new approach that combines factor graph optimization (FGO) and the maximum entropy criterion is proposed. This method replaces conventional nonlinear filters, whose performance degrades in the case of non-Gaussian measurement noise [2]. It also incorporates USBL into INS/DVL navigation. Another approach that also uses FGO and fuses USBL, INS, and DVL is proposed [3]. In [3], a robust M-estimation algorithm is incorporated into FGO. To improve INS/DVL integrated navigation performance when the measurements are corrupted by heavy-tailed non-Gaussian noise, a method that incorporates the error state Kalman filter (ESKF) with the interactive multiple model (IMM) is proposed [5]. Many of the previous methods concentrate on fusing the sensors; however, they often ignore the misalignment of sensors.

The misalignment of INS and DVL indicates the rotation of the DVL coordinate frame relative to the INS coordinate frame. In INS/DVL fused navigation, the velocity measured by the DVL is converted to the velocity in the INS coordinate frame using the rotation data of the DVL coordinate frame relative to the INS coordinate frame. If the coordinate frames of the INS and DVL are the same, the velocity measured by the DVL is identical to the velocity relative to the INS coordinate frame Any unknown misalignment between these two coordinate frames causes velocity errors, resulting in navigation errors. The degraded navigation performance affects the operational accuracy and stability of underwater vehicles during missions. Additionally, in a complex underwater environment with disturbances such as currents, waves, and tidal changes, the sensor output is affected to a greater degree; thus, the error increases further, and INS/DVL fused navigation deteriorates. In our research, rotation and attitude have the same kind of data representation and will be used interchangeably in the context of arithmetic operations.

Several approaches to evaluate the misalignment of sensors have been proposed. Ref. [6] evaluated the misalignment using the apparent velocity model and KF. Ref. [7] also estimates the misalignment using KF based on an approximated linear model. Ref. [4] proposed the arctangent fading memorial sage-husa adaptive Kalman filter (SHAKF) approach for evaluating misalignment. Ref. [8] estimated the misalignment using ESKF and incorporated Lie theory. In deriving the ESKF, attitude error is approximated linearly [8]. These approaches determine the misalignment before the operation of the underwater vehicle and are not applicable to online real-time operation They do not provide simultaneous navigation results. In addition, the nonlinear nature of the rotation is not fully addressed, even when Lie theory is employed. These methods do not handle operations on attitude or rotation rigorously, thus they result in slow convergence and do not guarantee convergence of the estimation depending on the circumstances.

The arithmetic operations on the attitude and misalignment, including differentiation, integration, and covariance calculations required to implement KF methodology are not trivial since attitude does not belong in a linear space [9,10,11]. Attitude and misalignment belong to nonlinear spaces, such as the 3-dimensional special orthogonal group SO(3) and 3-sphere space $S^{3}$ [12,13]. The research [14,15] used usual operations on attitude assuming that linear approximation does not cause a big problem. Instead, they applied additional operations, such as normalization for approximation [16] or optimization of attitude using the Levenberg-Marquardt algorithm [17], followed by operations on the attitude. However, these approximations eventually impair the reliability and stability of the estimation. To solve this problem, we incorporate Lie theory into a KF to estimate the misalignment between sensors as well as the pose(position and attitude) of an underwater vehicle. Specifically, the extended KF (EKF) is implemented in the Lie groups of 3-dimensional special Euclidean group SE(3) and $S^{3}$.

The Lie-theory-based approach can also be applied to the particle filter (PF), cubature KF (CKF), and unscented KF (UKF) [18,19,20,21,22]. To incorporate Lie theory into PF, CKF, and UKF, mathematical transformations are required to render the approach compatible with Lie theory. The mathematical transformations involve additional calculations, which increase the computational load and time in the estimation process. The additional calculations are mostly for exponential and logarithmic functions for the addition and subtraction of particles and sigma points [18,20]. A method utilizing an optimal regression matrix was proposed to reduce the computational complexity [21].

Recently, several researchers employed Lie theory explicitly or implicitly for KF applications for underwater navigation [8,23,24]. Lie theory combines strap-down INS (SINS) and DVL to solve the initial alignment problem for underwater vehicle navigation [8]. Another method, the Invariant Extended Kalman Filter (IEKF), extends the EKF by incorporating the characteristics of error dynamics, which are represented by a log-linear differential equation for underwater vehicle navigation [23]. A Lie group EKF is also beneficial for the heading initialization of autonomous underwater robots in situations with magnetic disturbance [24]. Whereas previous studies utilizing Lie theory approaches employed a theoretical approach using only a single Lie group space, our research incorporates two Lie groups SE(3) and $S^{3}$ into a single state vector. This makes it possible to estimate both the pose of the vehicle and the misalignment of sensors simultaneously.

One of the new features of this research is the use of Lie theory for the simultaneous estimation of the position and attitude of a vehicle as well as the misalignment. Also, the use of the two Lie groups in one state vector is new feature of this research. The use of the Lie theory facilitates the differentiation of the process model and measurement model to derive the Jacobian matrices, which are essential for EKF implementation. The proposed method follows the principle of IEKF and the stability and convergence of the approach is well established [25]. Though the Lie theory based implementation is not trivial unlike the Kalman filter implementation in a linear space, the performance of the methodology is proved in industrial implementations [26]. It results in accurate and consistent estimation which is robust against disturbances [27].

The novelty and contributions of the proposed approach are summarized as follows:

1. A method for numerically calculating Jacobians using Lie theory is introduced. This approach alleviates the need for analytical derivation of Jacobians, simplifying the implementation of the Kalman filter, especially in cases where the process and measurement models are complex.
2. It proposes an estimation method that uses state variables that belong in different Lie groups. It demonstrates that in cases where diverse Lie groups are combined for state variables, the Lie theory applies coherently, provided that operations corresponding to each Lie group are consistently employed.
3. To the best of our knowledge, this research is the first to propose the use of Lie theory for the explicit estimation of misalignment, along with navigation variables such as position, attitude, and velocity. The application of Lie theory enhances estimation performance by reducing the estimation error and improving the convergence of the Kalman gain and measurement innovation.
4. Two different Lie group elements in SE(3) and $S^{3}$ are incorporated into a state vector. This enables all the operations on the state variables and measurement variables to be rigorous without approximation. This proves that even though different Lie groups have different Lie algebras, they can be treated by a unified approach and simultaneously by Lie theory.
5. The procedure to find the Jacobian matrices of the process model and measurement model in the Lie group is described in detail, thus it shows how the Lie theory is applied in physical implementation.
6. This paper demonstrates the details involved in the implementation, and provides easy-to-follow guidance on the use of Lie theory for estimation.

The remainder of this paper is organized as follows. Section 2 describes the process and measurement models of the estimator based on the Lie theory. Section 3 presents the implementation of the EKF in the framework of Lie theory, using the Lie groups SE(3) and $S^{3}$. It includes the derivation of Jacobian matrices utilizing proper Lie theory. Section 4 describes the simulation results, and verifies the improvements made by the proposed method through comparisons with two other methods. One of the other methods neither uses Lie theory nor compensates for the misalignment. The second method estimates and compensates for the misalignment, but does not use Lie theory. Section 5 verifies the proposed method through field experiments that use a remotely operated vehicle (ROV). Section 6 concludes the paper and suggests directions for future research.

## 2. Process and Measurement Models Using the Lie Theory

This section begins with a definition of misalignment, a parameter that will be estimated. Then, essential tools from Lie theory, commonly used in applications to the KF, will be provided. Finally, the models will be derived using these tools.

### 2.1. Misalignment

**Figure 1:**
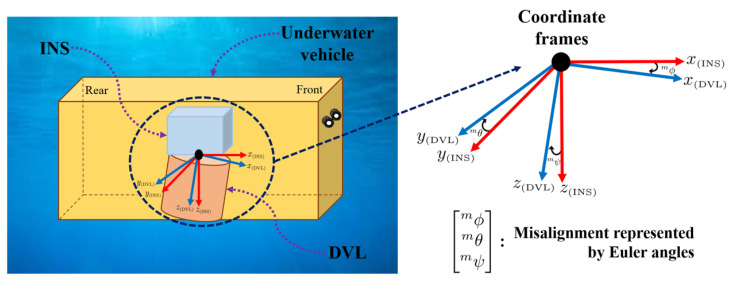
Misalignment indicates the rotation of the DVL coordinate frame relative to the INS coordinate frame.

In this study, the position and attitude of the underwater vehicle were considered to be the position and attitude of the INS. This indicates that the INS coordinate frame coincides with the coordinate frame of the underwater vehicle. The attitude of DVL is assumed to be the same as the INS. Therefore, misalignment indicates the rotation of DVL relative to the INS as shown in Figure 1.

The assumption that the DVL coordinate frame is the same as the INS coordinate frame can be applied generally even for the cases where the DVL is mounted with a predefined rotation relative to the INS coordinate frame. The predefined rotation can transform the velocity measured by the DVL to the velocity of the virtual DVL whose coordinate frame coincides with the INS coordinate frame, in compliance with the assumption. In this case, misalignment refers to the misalignment between the actual rotation and the predefined rotation.

### 2.2. Lie Theory Tools for KF

The position and attitude of an underwater vehicle compose the Lie group SE(3) and the unit quaternions representing the misalignment compose the Lie group $S^{3}$ [11]. The increment and difference of Lie group elements in SE(3) and $S^{3}$ belong in Lie algebra se(3) and $s^{3}$ respectively. The Lie algebra se(3) and $s^{3}$ correspond to the Lie group SE(3) and $S^{3}$, respectively. Lie theory uses exponential function and logarithmic function to calculate the difference between two Lie group elements and to incorporate increment to a Lie group element. In addition, differentiation and integration are defined by using the exponential function and logarithmic function. Therefore, Lie theory is vital for the implementation of KF in the following stages: state prediction by integration, calculation of measurement innovation, representation of covariance matrix, correction of predicted state by incorporating the measurement innovation, and so on. The Lie theory approach enables the computation of the state error, noise, and increments without linear approximation.

#### 2.2.1. Exponential Function

Let M be a Lie group and m be the Lie algebra corresponding to the Lie group M. The function Exp(τ) maps a Lie algebra element τ∈m to a Lie group element χ∈M. Exp(τ) differs from group to group [11]. If τ∈se(3), then Exp(τ) is as follows.

(1) $$\begin{array}{cc} \; & \mathrm{Exp}\left(\tau \right)=\left[\begin{array}{cc} \mathrm{Exp}(\theta ) & V(\theta )\rho \\ 0_{1\times 3} & 1 \end{array}\right]\in \mathrm{SE}\left(3\right),\;\tau =\left[\begin{array}{c} \rho \\ \theta \end{array}\right]\in \mathrm{se}\left(3\right). \end{array}$$

V(θ) and Exp(θ) are calculated as

(2) $$\begin{array}{cc} \; & V\left(\theta \right)=I+\frac{1-\cos(\theta )}{\theta }{\left[u\right]}_{\times }+\frac{\theta -\sin(\theta )}{\theta }{\left[u\right]}_{\times }^{2},\\ \; & \mathrm{Exp}\left(\theta \right)=I+\sin\left(\theta \right){\left[u\right]}_{\times }+\left(1-\cos\left(\theta \right)\right){\left[u\right]}_{\times }^{2}, \end{array}$$

where, θ=θu,∥u∥=1, and ${\left[\begin{array}{c} \cdot \end{array}\right]}_{\times }$ is an operator that transforms a vector into a skew-symmetric matrix.

If $\tau \in s^{3}$, then Exp(τ) is as follows.

(3) $$\begin{array}{cc} \; & \mathrm{Exp}\left(\tau \right)=\left[\begin{array}{c} \cos\left(\frac{\theta }{2}\right)\\ u\cdot \sin\left(\frac{\theta }{2}\right) \end{array}\right]\in S^{3},\;\tau =\theta u\in s^{3},\;∥u∥=1. \end{array}$$

#### 2.2.2. Logarithmic Function

The function Log(χ) maps a Lie group element χ∈M to a Lie algebra element τ∈m. It is the inverse map of Exp(τ). Log(χ) differs from group to group [11]. If χ∈SE(3), then Log(χ) is as follows.

(4) $$\mathrm{Log}\left(\chi \right)=\left[\begin{array}{c} V^{-1}\left(\theta \right)p\\ \mathrm{Log}(R) \end{array}\right]\in \mathrm{se}\left(3\right),\;\chi =\left[\begin{array}{cc} R & p\\ 0_{1\times 3} & 1 \end{array}\right]\in \mathrm{SE}\left(3\right).$$

In Equation (4), Log(R) and θ satisfies the followings:

(5) Log(R)=θ=θu

(6) $$\theta =\arccos\left(\frac{\mathrm{trace}(R)-1}{2}\right)$$

(7) $$u=\frac{{\left(R-R^{T}\right)}^{\vee }}{2\sin(\theta )}.$$

In Equation (7), ${\left(\cdot \right)}^{\vee }$ is an operator that transforms a skew-symmetric matrix into a vector [10,11].

If $\chi \in S^{3}$, then Log(χ) is as follows.

(8) $$\mathrm{Log}\left(\chi \right)=\theta =\theta u\in s^{3},\;\chi ={\left[\begin{array}{cccc} q_{w} & q_{x} & q_{y} & q_{z} \end{array}\right]}^{T}\in S^{3},$$

where,

(9) $$\begin{array}{cc} \chi & ={\left[\begin{array}{cccc} q_{w} & q_{x} & q_{y} & q_{z} \end{array}\right]}^{T}={\left[\begin{array}{cc} q_{w} & q_{v} \end{array}\right]}^{T}\\ \theta & =2\cdot \mathrm{atan}2(∥q_{v}∥,q_{w})\\ u & =\frac{q_{v}}{∥q_{v}∥}. \end{array}$$

#### 2.2.3. Plus and Minus Operator

The operator ⊕ combines a Lie group element χ∈M with a Lie algebra element τ∈m, resulting in a Lie group element in M. Conversely, the operator ⊖ calculates the difference between two Lie group elements $\chi_{1}$ and $\chi_{2}$ in a Lie group M, yielding a Lie algebra element in m.

The operator ⊕ is derived using the function Exp(·) as follows.

(10) $$\begin{array}{c} \chi ⊕\tau =\chi \cdot \mathrm{Exp}(\tau )\in M. \end{array}$$

The operator ⊖ is derived using the function Log(·) as follows.

(11) $$\begin{array}{c} \chi_{1}⊖\chi_{2}=\mathrm{Log}\left(\chi_{2}^{-1}\cdot \chi_{1}\right)\in m. \end{array}$$

These derivations hold regardless of the Lie group M and its corresponding Lie algebra m pair (M,m), provided that the functions Exp(·) and Log(·) are used consistently depending on the pair (M,m) [10,11].

### 2.3. Process Model

The state vector X(t) at time *t* consists of the position and attitude M(t) of the vehicle and the misalignment ^*m*^**q**(*t*) as follows:

(12) $$X\left(t\right)={\left[\begin{array}{cc} M(t) & {\;}^{m}q\left(t\right) \end{array}\right]}^{T}.$$

In Equation (12), M(t) is a 4 × 4 matrix that represents the position and attitude of an underwater vehicle which composes the Lie group SE(3) as follows:

(13) $$M\left(t\right)=\left[\begin{array}{cc} R(q(t)) & p(t)\\ 0_{1\times 3} & 1 \end{array}\right]\in \mathrm{SE}\left(3\right).$$

In Equation (13), $\;p\left(t\right)={\left[\begin{array}{ccc} x(t) & y(t) & z(t) \end{array}\right]}^{T}$ represents the position of the vehicle which belongs in three-dimensional real vector space. R(q(t)) is the rotation matrix corresponding to the unit quaternion q(t). The position is described in the North-East-Down (NED) coordinate frame. $q\left(t\right)={\left[\begin{array}{cccc} q_{w}\left(t\right) & q_{x}\left(t\right) & q_{y}\left(t\right) & q_{z}\left(t\right) \end{array}\right]}^{T}$ is the attitude of the underwater vehicle represented by a unit quaternion. In Equation (12), ^*m*^**q**(*t*) is the misalignment, which is the rotation of the true DVL coordinate frame from the INS coordinate frame. Because the rotations constitute the Lie group $S^{3}$, ^*m*^**q**(*t*) belongs to $S^{3}$.

(14) $${\;}^{m}q\left(t\right)={\left[\begin{array}{cccc} {\;}^{m}q_{w}\left(t\right) & {\;}^{m}q_{x}\left(t\right) & {\;}^{m}q_{y}\left(t\right) & {\;}^{m}q_{z}\left(t\right) \end{array}\right]}^{T}\in S^{3}.$$

To describe both the attitude and position in one frame, SE(3) is used. SE(3) contains the Lie group SO(3) to describe the attitude. The attitude of the vehicle q(t) converts to the rotation matrix in SO(3) as the following.

(15) $$\begin{array}{ccc} \; & R(q(t))=\; & \left[\begin{array}{ccc} 1-2q_{y}^{2}\left(t\right)-2q_{z}^{2}\left(t\right) & 2q_{x}\left(t\right)q_{y}\left(t\right)-2q_{z}\left(t\right)q_{w}\left(t\right) & 2q_{x}\left(t\right)q_{z}\left(t\right)+2q_{y}\left(t\right)q_{w}\left(t\right)\\ 2q_{x}\left(t\right)q_{y}\left(t\right)+2q_{z}\left(t\right)q_{w}\left(t\right) & 1-2q_{x}^{2}\left(t\right)-2q_{z}^{2}\left(t\right) & 2q_{y}\left(t\right)q_{z}\left(t\right)-2q_{x}\left(t\right)q_{w}\left(t\right)\\ 2q_{x}\left(t\right)q_{z}\left(t\right)-2q_{y}\left(t\right)q_{w}\left(t\right) & 2q_{x}\left(t\right)q_{w}\left(t\right)+2q_{y}\left(t\right)q_{z}\left(t\right) & 1-2q_{x}^{2}\left(t\right)-2q_{y}^{2}\left(t\right) \end{array}\right]. \end{array}$$

The process model describes state $X(t_{k+1})$ as a function of state $X(t_{k})$ and the measured input $U(t_{k})$ as follows:

(16) $$\begin{array}{cc} \; & X\left(t_{k+1}\right)=f\left(X\left(t_{k}\right),U\left(t_{k}\right)\right)+w,\;w∼N\left(0,Q\right), \end{array}$$

where w is the process noise. $U(t_{k})$ is the measured input which is expressed as follows:

(17) $$U\left(t_{k}\right)=\left[\begin{array}{c} v(t_{k})\\ \omega (t_{k}) \end{array}\right].$$

In Equation (17), $v\left(t_{k}\right)={\left[\begin{array}{ccc} u(t_{k}) & v(t_{k}) & w(t_{k}) \end{array}\right]}^{T}$ is the linear velocity of the vehicle. $\omega \left(t_{k}\right)={\left[\begin{array}{ccc} p(t_{k}) & q(t_{k}) & r(t_{k}) \end{array}\right]}^{T}$ is the angular velocity measured by INS.

Lie theory provides the process model for the location and attitude $M(t_{k})$ of the vehicle as Equation (18).

(18) $$\begin{array}{cc} M(t_{k+1}) & =M\left(t_{k}\right)\;⊕\;\left(U\left(t_{k}\right)\cdot ▵t_{k+1}\right), \end{array}$$

where $▵t_{k+1}$ denotes $t_{k+1}-t_{k}$. The operator ⊕ which is implemented by using the exponential function adds a Lie algebra element to a Lie group element.

The measured velocity ^*D*^**v**(*t_k_*) should be transformed to the velocity $v(t_{k})$ in the INS coordinate frame using the misalignment ${\;}^{m}q\left(t_{k}\right)$, as follows:

(19) $$v\left(t_{k}\right)={\;}^{m}q\left(t_{k}\right)⊗{\;}^{D}v\left(t_{k}\right)⊗{\;}^{m}q^{*}\left(t_{k}\right),$$

where ${\;}^{D}v\left(t_{k}\right)$ is the linear velocity measured by the DVL and ⊗ is the operator that multiplies two quaternions [10].

In Equation (10), $M(t_{k})$ is in Lie group SE(3) and $U\left(t_{k}\right)\cdot ▵t_{k+1}$ is in Lie algebra se(3) which is associated with SE(3). $U\left(t_{k}\right)\cdot ▵t_{k+1}$ indicates changes in the 6 degrees of freedom motion and is represented as a six-dimensional vector in $R^{6}$.

(20) $$\begin{array}{c} U\left(t_{k}\right)\cdot ▵t_{k+1}=\left[\begin{array}{c} ▵t_{k+1}\cdot v\left(t_{k}\right)\\ ▵t_{k+1}\cdot \omega \left(t_{k}\right) \end{array}\right]=\left[\begin{array}{c} \rho \\ \theta \end{array}\right]\in R^{6}. \end{array}$$

The misalignment is assumed to be constant over time because the sensors are mechanically fixed to the underwater vehicle. Therefore, the process model for the misalignment ${\;}^{m}q\left(t_{k}\right)$ is as follows:

(21) $${\;}^{m}q\left(t_{k+1}\right)={\;}^{m}q\left(t_{k}\right).$$

The function $f(X\left(t_{k}\right),U\left(t_{k}\right))$ in the process model of Equation (16) for EKF implementation comprises Equations (18) and (21). Equation (18), together with Equation (19), compensates for misalignment and updates the state. By utilizing Lie theory, exact state prediction without approximation errors is made possible.

### 2.4. Measurement Model

The position of the underwater vehicle measured by the Ultra-Short Baseline (USBL) and the attitude by the INS are used as the measurements for the estimation [28]. The measurement vector Z(t) is represented as a Lie group comprising position and attitude as follows:

(22) $$Z\left(t\right)=\left[\begin{array}{cc} R(\tilde{q}\left(t\right)) & \tilde{p}\left(t\right)\\ 0_{1\times 3} & 1 \end{array}\right]\in \mathrm{SE}\left(3\right),$$

(23) $$\mathrm{where},\;\tilde{p}\left(t\right)=\left[\begin{array}{c} \tilde{x}\left(t\right)\\ \tilde{y}\left(t\right)\\ \tilde{z}\left(t\right) \end{array}\right];\;\tilde{q}\left(t\right)=\left[\begin{array}{c} {\tilde{q}}_{w}\left(t\right)\\ {\tilde{q}}_{x}\left(t\right)\\ {\tilde{q}}_{y}\left(t\right)\\ {\tilde{q}}_{z}\left(t\right) \end{array}\right].$$

In Equation (22), $\tilde{p}\left(t\right)$ is the position measured by the USBL and $\tilde{q}\left(t\right)$ is the attitude measured by INS. The rotation matrix $R(\tilde{q}\left(t\right))\in SO\left(3\right)$ is computed using Equation (15). The measurements Z(t) constitute the Lie group *SE*(3).

The measurement model is a function which relates the state $X(t_{k})$ to the measurement $Z(t_{k})$, as follows:

(24) $$\begin{array}{cc} Z(t_{k})= & h(X\left(t_{k}\right))+n\\ = & M\left(t_{k}\right)+n,\;n∼N\left(0,R\right), \end{array}$$

where n is the measurement noise, which is a Gaussian distributed random variable with a mean of zero and covariance R.

## 3. Use of Lie Theory for Estimation in Lie Groups SE(3) and $S^{3}$

Lie theory is needed for operations on states and measurements which are required for implementation of EKF. The process model and measurement model are linearized to Jacobian matrices by partial differentiation of the models. The partial differentiation requires Lie theory. Also, adding increments to the states and calculating the difference between state variables requires Lie theory. If Lie theory is not utilized, the operations involve errors, and this deteriorates the estimation accuracy and convergence performance. This section explains the use of Lie theory for the derivation of Jacobian matrices and arithmetic operations.

### 3.1. Jacobian of Process Model Relative to State Variables

The Jacobian matrix of the process model Equations (18) and (21) with respect to state variables is given by

(25) $$\begin{array}{cc} A(t_{k+1}) & =\left[\begin{array}{cc} \frac{\partial M(t_{k+1})}{\partial M(t_{k})} & \frac{\partial M(t_{k+1})}{\partial^{m}q\left(t_{k}\right)}\\ \frac{\partial^{m}q\left(t_{k+1}\right)}{\partial M(t_{k})} & \frac{\partial^{m}q\left(t_{k+1}\right)}{\partial^{m}q\left(t_{k}\right)} \end{array}\right]\\ \; & =\left[\begin{array}{cccc} A_{1}\left(t_{k+1}\right) & A_{2}\left(t_{k+1}\right) & \cdots & A_{9}\left(t_{k+1}\right) \end{array}\right], \end{array}$$

where $A(t_{k+1})$ has the size of 9 × 9. $A(t_{k+1})$ is numerically computed through the finite difference method based on the Lie theory as

(26) $$\begin{array}{cc} A_{i}\left(t_{k+1}\right) & ≃\frac{f\left(X\left(t_{k}\right)⊕\delta_{i},U\left(t_{k}\right)\right)⊖f\left(X\left(t_{k}\right),U\left(t_{k}\right)\right)}{\delta }, \end{array}$$

where the operator ⊖ returns a Lie algebra element which represents the difference between two Lie group elements. δ should be set to a sufficiently small value, and it is taken into the vector $\delta_{i}$ when the operation is performed as many times as the sum of the dimension of the Lie algebras se(3) and $s^{3}$.

(27) $$\delta_{1}=\left[\begin{array}{c} \delta \\ 0\\ 0\\ 0\\ 0\\ 0\\ 0\\ 0\\ 0 \end{array}\right],\;\delta_{2}=\left[\begin{array}{c} 0\\ \delta \\ 0\\ 0\\ 0\\ 0\\ 0\\ 0\\ 0 \end{array}\right],\;\cdots ,\;\delta_{9}=\left[\begin{array}{c} 0\\ 0\\ 0\\ 0\\ 0\\ 0\\ 0\\ 0\\ \delta \end{array}\right].$$

The calculation of $A(t_{k+1})$ requires different procedures depending on the Lie groups SE(3) and $S^{3}$. This is because the state $X(t_{k})$ contains both the elements of SE(3) and $S^{3}$. Although the element of SE(3) has 4×4 components, the increments form a 6-dimensional tangent space se(3). Likewise, while the unit quaternions in $S^{3}$ has four components, its increments are in a 3-dimensional tangent space $s^{3}$.

$A_{i}\left(t_{k+1}\right)$(1:6) which is part of $A_{i}\left(t_{k+1}\right)$ is calculated as follows:

(28) $$\begin{array}{cc} \; & A_{i}\left(t_{k+1}\right)\left(1:6\right)=\left[\begin{array}{cc} \frac{\partial M(t_{k+1})}{\partial M(t_{k})} & \frac{\partial M(t_{k+1})}{\partial^{m}q\left(t_{k}\right)} \end{array}\right]\\ \; & ≃\frac{f\left(M\left(t_{k}\right)⊕\delta_{i}\left(1:6\right),U\left(t_{k}\right)\right)⊖f\left(M\left(t_{k}\right),U\left(t_{k}\right)\right)}{\delta }. \end{array}$$

The vector $\delta_{i}$(1:6) which comprises the first 6 rows of $\delta_{i}$ is the vector corresponding to an element in the Lie algebra se(3). Expanding $M\left(t_{k}\right)⊕\delta_{i}$(1:6) yields $M(t_{k})$·Exp ($\delta_{i}$(1:6)). We employ Equation (1) for the computation of Exp(·) function.

Next, the remaining part of the Jacobian of $A_{i}\left(t_{k+1}\right)$ is calculated as follows:

(29) $$\begin{array}{cc} \; & A_{i}\left(t_{k+1}\right)\left(7:9\right)=\left[\begin{array}{cc} \frac{\partial^{m}q\left(t_{k+1}\right)}{\partial M(t_{k})} & \frac{\partial^{m}q\left(t_{k+1}\right)}{\partial^{m}q\left(t_{k}\right)} \end{array}\right]\\ \; & ≃\frac{f\left({\;}^{m}q\left(t_{k}\right)⊕\delta_{i}\left(7:9\right),U\left(t_{k}\right)\right)⊖f\left({\;}^{m}q\left(t_{k}\right),U\left(t_{k}\right)\right)}{\delta }. \end{array}$$

In Equation (29), $\delta_{i}\left(7:9\right)$ which comprises rows (7:9) of $\delta_{i}$ is the tangent vector in Lie algebra $s^{3}$. Expanding ${\;}^{m}q\left(t_{k}\right)⊕\delta_{i}$(7:9) yields ${\;}^{m}q\left(t_{k}\right)$⊗Exp($\delta_{i}$(7:9)).

The Jacobian matrix $A_{i}\left(t_{k+1}\right)\left(7:9\right)$ can be calculated numerically using Equation (29). Alternatively, $A_{i}\left(t_{k+1}\right)\left(7:9\right)$ can be derived analytically as follows:

(30) $$\frac{\partial^{m}q\left(t_{k+1}\right)}{\partial M(t_{k})}=0_{3\times 6},\;\frac{\partial^{m}q\left(t_{k+1}\right)}{\partial^{m}q\left(t_{k}\right)}=I_{3}.$$

### 3.2. Jacobian of Process Model with Respect to Measured Input Variables

The Jacobian matrix for Equation (17) with respect to the measured input is as follows:

(31) $$\begin{array}{cc} {\;}^{U}A\left(t_{k+1}\right) & =\left[\begin{array}{cc} \frac{\partial M(t_{k+1})}{\partial v(t_{k})} & \frac{\partial M(t_{k+1})}{\partial \omega (t_{k})}\\ \frac{\partial^{m}q\left(t_{k+1}\right)}{\partial v(t_{k})} & \frac{\partial^{m}q\left(t_{k+1}\right)}{\partial \omega (t_{k})} \end{array}\right]\\ \; & =\left[\begin{array}{cccc} {\;}^{U}A_{1}\left(t_{k+1}\right) & {\;}^{U}A_{2}\left(t_{k+1}\right) & \cdots & {\;}^{U}A_{6}\left(t_{k+1}\right) \end{array}\right], \end{array}$$

where ${\;}^{U}A\left(t_{k+1}\right)$ is a 9×6 matrix. The first six rows of ${\;}^{U}A\left(t_{k+1}\right)$ can be calculated numeriacally by Equation (32).

(32) $$\begin{array}{cc} {\;}^{U}A_{i}\left(t_{k+1}\right)\left(1:6\right) & =\left[\begin{array}{cc} \frac{\partial M(t_{k+1})}{\partial v(t_{k})} & \frac{\partial M(t_{k+1})}{\partial \omega (t_{k})} \end{array}\right]\\ \; & ≃\frac{f\left(M\left(t_{k}\right),U\left(t_{k}\right)+\epsilon_{i}\right)⊖f\left(M\left(t_{k}\right),U\left(t_{k}\right)\right)}{\epsilon }. \end{array}$$

Similar to $\delta_{i}$ and δ in Equation (26), ϵ has a sufficiently small value, and it is taken into the vector $\epsilon_{i}$ when the operation is performed 6 times.

(33) $$\epsilon_{1}=\left[\begin{array}{c} \epsilon \\ 0\\ 0\\ 0\\ 0\\ 0 \end{array}\right],\;\epsilon_{2}=\left[\begin{array}{c} 0\\ \epsilon \\ 0\\ 0\\ 0\\ 0 \end{array}\right],\;\cdots ,\;\epsilon_{6}=\left[\begin{array}{c} 0\\ 0\\ 0\\ 0\\ 0\\ \epsilon \end{array}\right].$$

The last three rows of ${\;}^{U}A\left(t_{k+1}\right)$ are derived analytically as follows:

(34) $${\;}^{U}A_{i}\left(t_{k+1}\right)\left(7:9\right)=\left[\begin{array}{cc} \frac{\partial^{m}q\left(t_{k+1}\right)}{\partial v(t_{k})} & \frac{\partial^{m}q\left(t_{k+1}\right)}{\partial \omega (t_{k})} \end{array}\right]=\left[\begin{array}{cc} 0_{3} & 0_{3} \end{array}\right].$$

Because the misalignment ${\;}^{m}q\left(t_{k+1}\right)$ does not depend on both $v(t_{k})$ and $\omega (t_{k})$, the partial derivatives of ${\;}^{m}q\left(t_{k+1}\right)$ with respect to $v(t_{k})$ and $\omega (t_{k})$ are zeros, as shown in Equation (34).

### 3.3. Jacobian of Measurement Model

The Jacobian matrix for the measurement model in Equation (24) is derived as follows:

(35) $$H\left(t_{k}\right)=\frac{\partial h(X\left(t_{k}\right))}{\partial X(t_{k})}=\left[\begin{array}{cc} \frac{\partial M(t_{k})}{\partial M(t_{k})} & \frac{\partial M(t_{k})}{\partial^{m}q\left(t_{k}\right)} \end{array}\right]=\left[\begin{array}{cc} I_{6} & 0_{6\times 3} \end{array}\right].$$

### 3.4. Lie Theory Based EKF Procedure

The EKF estimates position, attitude, and misalignment. It uses Jacobian matrices derived in Section 3.1, Section 3.2 and Section 3.3 using Lie theory. In addition to employing Jacobian matrices, the EKF uses arithmetic operations based on Lie theory.

The estimated state $\hat{M}\left(t_{k}\right)$ and ${\;}^{m}\hat{q}\left(t_{k}\right)$ are propagated to the states $\hat{M}\left(t_{k+1}^{-}\right)$ and ${\;}^{m}\hat{q}\left(t_{k+1}^{-}\right)$ for the time $t=t_{k+1}$ using the Equations (18) and (21), as follows:

(36) $$\begin{array}{cc} \hat{M}\left(t_{k+1}^{-}\right) & =\hat{M}\left(t_{k}\right)\;\cdot \;\mathrm{Exp}\left(U\left(t_{k}\right)\cdot ▵t_{k+1}\right)\\ {\;}^{m}\hat{q}\left(t_{k+1}^{-}\right) & ={\;}^{m}\hat{q}\left(t_{k}\right). \end{array}$$

In Equation (36), $\mathrm{Exp}(U\left(t_{k}\right)\cdot ▵t_{k+1})$ for Lie group SE(3) is calculated using Equation (1). The Lie group exponential operation guarantees accurate prediction. In contrast, the numerical integration of the method [29] causes errors in the predicted position and attitude.

The covariance $P(t_{k+1}^{-})$ of the predicted state $\hat{X}\left(t_{k+1}^{-}\right)={\left[\begin{array}{cc} \hat{M}\left(t_{k+1}^{-}\right) & {\;}^{m}\hat{q}\left(t_{k+1}^{-}\right) \end{array}\right]}^{T}$ is computed using the Jacobian matrices A and ${\;}^{U}A$ as follows:

(37) $$\begin{array}{cc} P(t_{k+1}^{-})= & A\left(t_{k+1}\right)\cdot P\left(t_{k}\right)\cdot A^{T}\left(t_{k+1}\right)+{\;}^{U}A\left(t_{k+1}\right)\cdot {\;}^{U}Q\cdot {\;}^{U}A^{T}\left(t_{k+1}\right)+Q. \end{array}$$

The covariance matrices $P(t_{k})$ and $P(t_{k+1}^{-})$ have a dimension of 9×9. This is a distinct characteristic of the Lie theory based EKF implementation compared with the EKF implementation by [29]. In [29], the covariance of the state has a dimension of 11×11, because it regarded position as a variable in $R^{3}$, attitude as a variable in $R^{4}$, and misalignment as a variable in $R^{4}$, not in Lie groups. Moreover, in [29], operations on attitude and misalignment do not provide proper attitude and misalignment.

Uncertainty in the measured input affects the estimation. This is considered by including ${\;}^{U}A\left(t_{k+1}\right)\cdot {\;}^{U}Q\cdot {\;}^{U}A^{T}\left(t_{k+1}\right)$ in Equation (37). Here, ${\;}^{U}Q$ is the covariance of the measured input and is a 6×6 matrix.

According to Lie theory, the measurement innovation is computed by using Log(·) function of Lie group SE(3) as follows:

(38) $$\begin{array}{cc} Y(t_{k+1}) & =Z\left(t_{k+1}\right)⊖h\left(\hat{X}\left(t_{k+1}^{-}\right),0\right)\\ \; & =\mathrm{Log}(h{\left(\hat{X}\left(t_{k+1}^{-}\right),0\right)}^{-1}\cdot Z\left(t_{k+1}\right))\\ \; & =\mathrm{Log}({\hat{M}}^{-1}\left(t_{k+1}^{-}\right)\cdot Z\left(t_{k+1}\right)). \end{array}$$

The Log(·) of the Lie group SE(3) is given as Equation (4). The value of Log(·) belongs in Lie algebra se(3).

The Kalman gain is computed as follows:

(39) $$\begin{array}{cc} \; & K\left(t_{k+1}\right)=P\left(t_{k+1}^{-}\right)\cdot H{\left(t_{k+1}\right)}^{T}\cdot S^{-1}\left(t_{k+1}\right)\\ \; & \mathrm{where},\;S\left(t_{k+1}\right)=H\left(t_{k+1}\right)\cdot P\left(t_{k+1}^{-}\right)\cdot H^{T}\left(t_{k+1}\right)+R. \end{array}$$

The Kalman gain $K(t_{k+1})$ is a 9×6 matrix.

Using Lie theory, the correction state $\delta \hat{X}\left(t_{k+1}\right)$ is added to the predicted state ${\hat{X}}^{-}\left(t_{k+1}\right)$ using the operator ⊕, to provide the corrected state $\hat{X}\left(t_{k+1}\right)$.

(40) $$\begin{array}{cc} \; & \hat{X}\left(t_{k+1}\right)=\hat{X}\left(t_{k+1}^{-}\right)⊕\delta \hat{X}\left(t_{k+1}\right)\\ \; & \mathrm{where},\;\delta \hat{X}\left(t_{k+1}\right)=\left[\begin{array}{c} \delta \hat{M}\left(t_{k+1}\right)\\ \delta^{m}\hat{q}\left(t_{k+1}\right) \end{array}\right]=K\left(t_{k+1}\right)\cdot Y \end{array}$$

Equation (40) is rephrased as,

(41) $$\begin{array}{c} \left[\begin{array}{c} \hat{M}\left(t_{k+1}\right)\\ {\;}^{m}\hat{q}\left(t_{k+1}\right) \end{array}\right]=\left[\begin{array}{c} \hat{M}\left(t_{k+1}^{-}\right)\\ {\;}^{m}\hat{q}\left(t_{k+1}^{-}\right) \end{array}\right]⊕\left[\begin{array}{c} \delta \hat{M}\left(t_{k+1}\right)\\ \delta^{m}\hat{q}\left(t_{k+1}\right) \end{array}\right] \end{array}$$

Lie theory implements Equation (41) using Exp(·) as follows:

(42) $$\begin{array}{cc} \hat{M}\left(t_{k+1}\right) & =\hat{M}\left(t_{k+1}^{-}\right)⊕\delta \hat{M}\left(t_{k+1}\right)=\hat{M}\left(t_{k+1}^{-}\right)\cdot \mathrm{Exp}\left(\delta \hat{M}\left(t_{k+1}\right)\right)\\ {\;}^{m}\hat{q}\left(t_{k+1}\right)\; & ={\;}^{m}\hat{q}\left(t_{k+1}^{-}\right)⊕\delta^{m}\hat{q}\left(t_{k+1}\right)={\;}^{m}\hat{q}\left(t_{k+1}^{-}\right)⊗\mathrm{Exp}\left(\delta^{m}\hat{q}\left(t_{k+1}\right)\right). \end{array}$$

In Equation (42), $\mathrm{Exp}(\delta \hat{M}\left(t_{k+1}\right))$ and $\mathrm{Exp}(\delta^{m}\hat{q}\left(t_{k+1}\right))$ are exponential functions in SE(3) and $S^{3}$, respectively, as shown in Equations (1) and (3). The measurement update using Equation (42) is accurate without approximation. If Lie theory is not applied, addition and subtraction for measurement update leads to improper results and approximation errors.

Finally, the predicted covariance $P(t_{k+1}^{-})$ is updated to the covariance $P(t_{k+1})$ of the estimated state $\hat{X}\left(t_{k+1}\right)$ as follows:

(43) $$\begin{array}{cc} P(t_{k+1})= & \left(I-K\left(t_{k+1}\right)\cdot H\left(t_{k+1}\right)\right)\cdot P\left(t_{k+1}^{-}\right)\cdot {\left(I-K\left(t_{k+1}\right)\cdot H\left(t_{k+1}\right)\right)}^{T}\\ \; & +K\left(t_{k+1}\right)\cdot R\cdot K^{T}\left(t_{k+1}\right). \end{array}$$

Equation (43) uses the Jacobian matrix $H(t_{k+1})$ which is derived using Lie theory as shown in Equation (35).

## 4. Simulation Test and Results

This section demonstrates the improvement achieved through the application of Lie theory via simulations. The proposed method is compared with the widely used quaternion-based EKF (Q-EKF) and the quaternion-based misalignment compensation EKF (QMC-EKF) [29]. While Q-EKF employs quaternion representation for attitude [30], it does not estimate or compensate for misalignment and does not utilize Lie theory. QMC-EKF estimates and compensates for the misalignment, and uses quaternions for attitude representation, However, it does not use Lie theory for the arithmetic operations.

The comparison focuses on two aspects: the accuracy of the state estimation results and the convergence property of internal variables. Estimation results refer to the estimated position, attitude, and misalignment. The internal variables represent measurement innovation and Kalman gain. Hereafter, the proposed approach is referred to as “Proposed” in the figures and tables, whereas “Q-EKF” and “QMC-EKF” denote the previous approaches, namely the quaternion based EKF and quaternion based misalignment compensation EKF, respectively.

### 4.1. Test under High Maneuverability Conditions

To clearly observe the improvements by the proposed method in the simulations, the underwater vehicle was assumed to perform highly dynamic three-dimensional maneuvers. Furthermore, to test the robustness of the proposed method, the noise, which is an unexpected perturbation, was added to the sensor data. The scenarios for the maneuver of the underwater vehicle and sensor noise are as follows:

- The initial position of the underwater vehicle is (10,10,30)(m), and the initial attitude is (0,0,0)(rad). The true misalignment of DVL from INS is (10°, −20°, 30°).
- The underwater vehicle maneuvers for 1200 s. The measurement rate of the INS and DVL is 10 Hz.
- The linear velocity of the underwater vehicle is $v\left(t\right)={\left[\begin{array}{ccc} 10 & 5 & 5 \end{array}\right]}^{T}\left(m/s\right)$ in the vehicle coordinate frame. The angular velocity is as follows.
  (44) $$\omega \left(t\right)=\left\{\begin{array}{cc} {\left[\begin{array}{ccc} -\frac{\pi }{5} & -\frac{\pi }{5} & -\frac{\pi }{5} \end{array}\right]}^{T}\left(\mathrm{rad}/s\right), & 0\;s\leq t<600\;s\\ {\left[\begin{array}{ccc} \frac{\pi }{5} & \frac{\pi }{5} & \frac{\pi }{5} \end{array}\right]}^{T}\left(\mathrm{rad}/s\right), & 600\;s\leq t<1200\;s \end{array}\right.$$
- The measurement data from the simulated sensors contain random noise. The measurement noise in the linear velocity provided by the DVL is $n_{v}∼N\left(0,0.2I_{3}\right)$. The measurement noise in the angular velocity provided by the INS is $n_{\omega }∼N\left(0,0.01I_{3}\right)$.
- Gaussian noise with the mean 0 and the standard deviation 0.7 m is applied to the measured position data in each coordinate, whereas Gaussian noise with the mean 0 and the standard deviation 0.03 rad is applied to the measured attitude in each coordinate.

**Figure 2:**
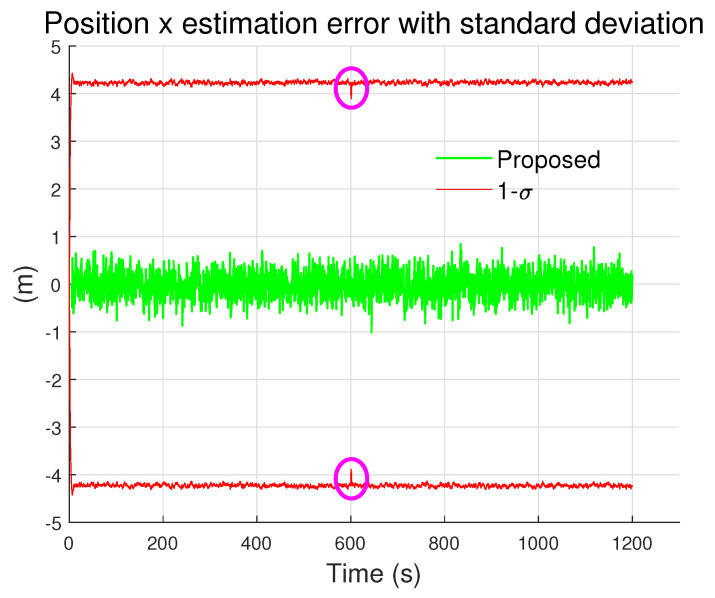
Position estimation error with standard deviation.

Figure 2 explains the robustness of the position estimation result. It displays the estimation error and standard deviation ($\sqrt{P\left(t_{k}\right)\left(1,1\right)}$) calculated by Equation (43) for the estimated x coordinate. The position estimation error remains within the standard deviation for the entire range. In addition, the standard deviation is kept within a bounded range, and does not diverge. At time t=600s, the angular velocity of the vehicle changes abruptly. Figure 2 shows that there is a noticeable change in the standard deviation at t=600s as indicated by the two circles. Nevertheless, the standard deviation returns to its normal value instantly. This indicates that the proposed method is stable and robust in dynamic maneuvering conditions.

**Figure 3:**
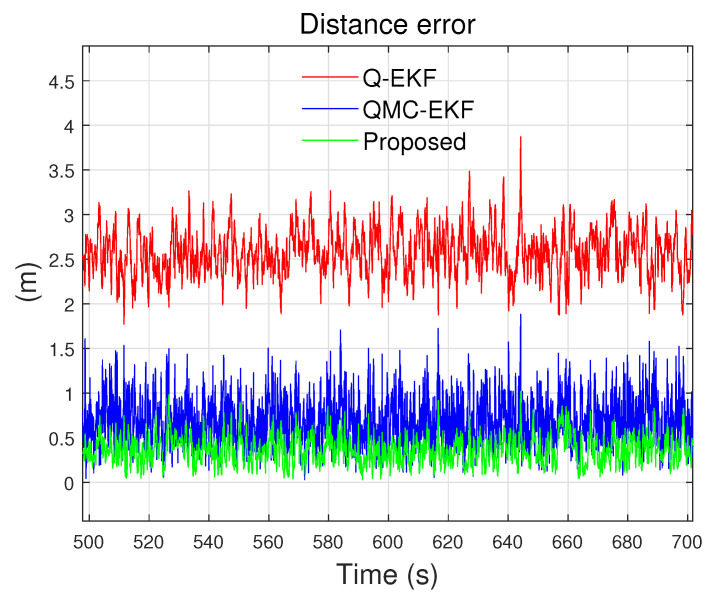
Comparison of distance error.

The error of the estimated position in the NED coordinate frame is compared to those of the Q-EKF and QMC-EKF, as shown in Figure 3. Here, the error of the estimated position is compared in terms of the distance error. The distance error refers to the distance between the estimated position $\hat{p}\left(t\right)={\left[\begin{array}{ccc} \hat{x}\left(t\right) & \hat{y}\left(t\right) & \hat{z}\left(t\right) \end{array}\right]}^{T}$ and the reference position $p\left(t\right)={\left[\begin{array}{ccc} x(t) & y(t) & z(t) \end{array}\right]}^{T}$ as defined by Equation (45).

(45) $$d\left(\hat{p}\left(t\right),p\left(t\right)\right)=\sqrt{{\left(\hat{x}\left(t\right)-x\left(t\right)\right)}^{2}+{\left(\hat{y}\left(t\right)-y\left(t\right)\right)}^{2}+{\left(\hat{z}\left(t\right)-z\left(t\right)\right)}^{2}}$$

The proposed method exhibits smaller errors than the others, the entire time. This performance improvement was attributed to the accurate Jacobian matrix calculation, the use of proper plus and minus operations, and rigorous covariance calculation.

**Figure 4:**
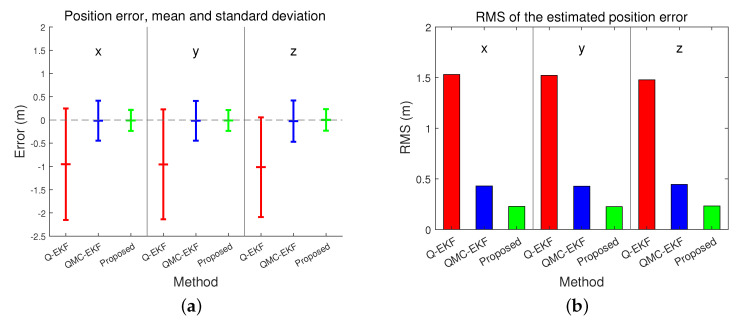
Comparison of errors in position estimation: (**a**) represents the mean and standard deviation of the estimated position error; and (**b**) indicates the RMS of the estimated position error.

**Figure 5:**
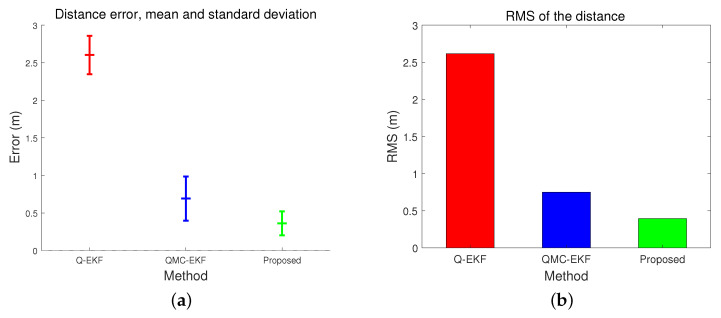
Comparison of distance error: (**a**) represents the mean and standard deviation of the distance error; and (**b**) indicates the RMS of the distance error.

The position estimation performance is compared in Table 1 and Table 2. Table 1 compares the position estimation errors in each coordinate, and Table 2 compares the distance error. Position errors in *x*, *y*, and *z* indicate $\left(\hat{x}\left(t\right)-x\left(t\right)\right)$, $\left(\hat{y}\left(t\right)-y\left(t\right)\right)$, and $\left(\hat{z}\left(t\right)-z\left(t\right)\right)$, respectively. Based on the data presented in Table 1 and Table 2, the position and distance errors are compared visually in Figure 4 and Figure 5, respectively. The proposed method significantly reduces the errors in terms of the mean, standard deviation, and Root Mean Square (RMS) for all three axes. The RMS in *x*-directional position error is calculated as follows.

(46) $$\sqrt{\frac{1}{N}\sum_{k=1}^{N}{\left(\hat{x}\left(t_{k}\right)-x\left(t_{k}\right)\right)}^{2}},$$

where *N* indicates the number of estimations. The mean of the position error in *x*-direction without compensation of misalignment (Q-EKF) is −0.9521 m, and it is improved to −0.0109 m by the proposed method. The RMS of *x*-direction error is improved to 0.2269 m by the proposed method from 1.5314 m by the Q-EKF. The results confirm that the proposed method estimates the trajectories more accurately than the other approaches.

**Figure 6:**
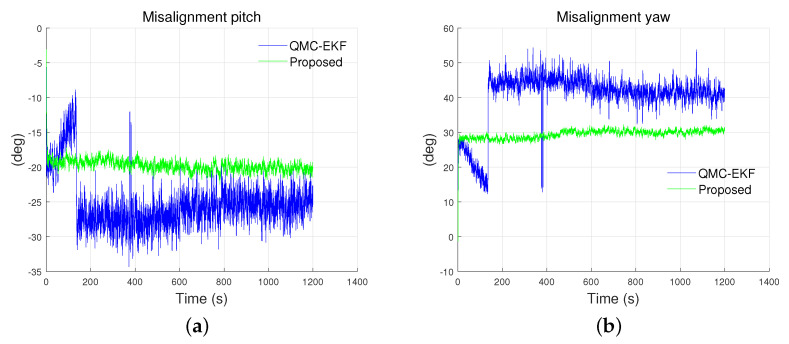
Misalignment estimated under the high maneuverability conditions: (**a**) pitch; (**b**) yaw. True misalignment for the pitch and yaw are −20° and 30°, respectively.

**Figure 7:**
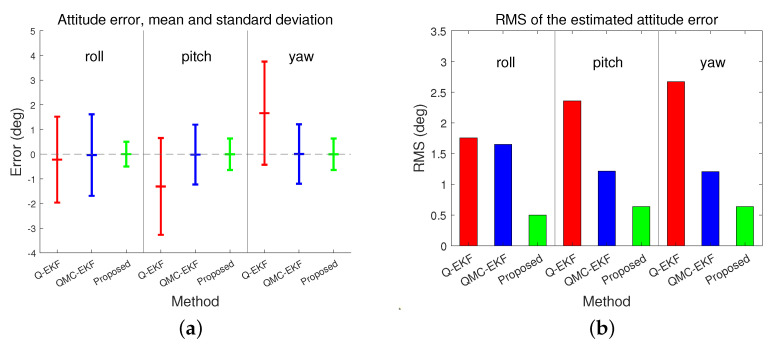
Comparison of errors in attitude estimation: (**a**) represents the attitude estimation error; and (**b**) indicates the RMS of the estimated attitude error.

**Figure 8:**
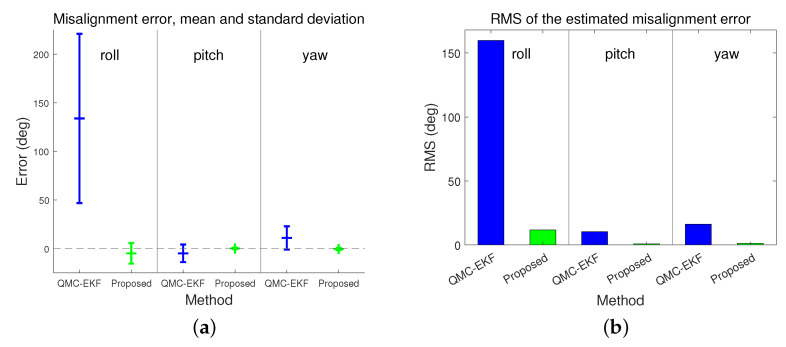
Comparison of errors in misalignment estimation: (**a**) represents the misalignment estimation error; and (**b**) indicates the RMS of the estimated misalignment error.

Figure 6 depicts the estimation of misalignments. The performance of the estimation for attitude and misalignment is shown in Table 3. The Q-EKF estimates the attitude of the underwater vehicle; however, it does not estimate the misalignment, and thus the statistics for Q-EKF corresponding to the misalignment are labeled as “Not Applicable (NA).” Figure 7 and Figure 8 present a graphical comparison of the errors in the estimation of attitude and misalignment based on Table 3. The proposed method exhibits improved estimation for both the attitude and misalignment. Unlike Q-EKF, QMC-EKF and the proposed method explicitly estimate the misalignment and compensate for the misalignment. However, QMC-EKF regards the attitude and the misalignment as the variables in a linear space. Therefore, QMC-EKF uses the usual arithmetic operations by which linear variables are treated. These operations result in approximations and require ad hoc normalizations. On the contrary, the proposed method uses Lie theory which provides rigorous arithmetic operations required to implement the Kalman filter. The improvements in estimation accuracy and stability conform to the theoretical verification [25] and are consistent with the examples shown in [26].

For QMC-EKF, the estimated misalignment has large errors, especially in the roll. If the vehicle moves in one of the principal axes, the misalignment in that direction does not affect the velocity measurement. Therefore, if the vehicle moves in the x-direction, the misalignment in roll is not distinctively perceivable by the velocity measurement. In the simulation, the x-directional velocity is twice the other directional velocities, and the estimation accuracy of roll misalignment is the worst of all. In addition to this effect, the approximations involved in QMC-EKF deteriorate the misalignment estimation in roll.

As shown in Figure 6 and Table 3, QMC-EKF exhibits unstable estimates in both pitch and yaw. On the contrary, the proposed method improves the estimation of misalignment and provides stable misalignment value. The proposed method estimates the misalignment in pitch as $-19.8314^{\circ }$, whereas QMC-EKF estimates it as $-24.9177^{\circ }$. As for the misalignment in yaw, it is estimated as $29.5798^{\circ }$ and $40.9918^{\circ }$, by the proposed method and QMC-EKF respectively. The proposed method exhibits improved estimation for both the attitude and misalignment. The proposed method not only reduces the mean error but also exhibits significant improvements in standard deviation and RMS errors.

The proposed method significantly improves the inherent properties of the estimation: Kalman gain and measurement innovation. The EKF based on the Lie theory, can model a nonlinear system more accurately. The linear approximation of arithmetic operations on rotation or attitude, which inherently exists in nonlinear space, necessitates ad hoc measures. As a remedy for this limitation, the use of Lie theory enables the rigorous application of the KF. Consequently, the convergence of the Kalman gain and measurement innovation as well as the covariance is improved.

Because the Lie theory provides accurate calculation of predicted measurement and the difference between two attitudes, measurement innovation becomes more accurate. Also, the covariance matrices represent the uncertainties more effectively. Therefore more appropriate calculation of Kalman gain is possible. This improves the convergence of the Kalman gain and measurement innovation.

**Figure 9:**
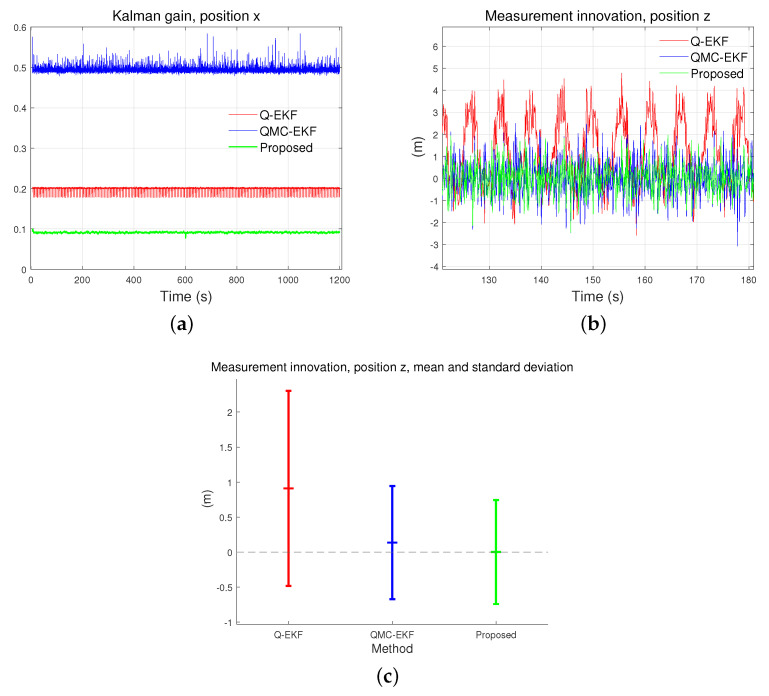
Comparison of Kalman gain and measurement innovation: (**a**) compares the Kalman gain for position x; (**b**) compares the measurement innovation for position z; and (**c**) compares the mean and standard deviation of measurement innovation for position z.

Figure 9 compares the Kalman gain and measurement innovation of the methods. They are calculated by using Equations (39) and (38). The Kalman gain and measurement innovation for the proposed method is lower than the others. Moreover, they exhibit lower variance than the others. Overall, the simulation results indicate that the proposed method outperforms the others in the accuracy and stability of estimation.

### 4.2. Test under Low Maneuverability Conditions

To demonstrate that the improvement by the proposed method is more significant in high maneuverability conditions, the proposed method is tested under low maneuverability conditions. The ROV moves at a velocity of (1, 0.5, 0.5) m/s, indicating lower maneuverability than the velocity (10, 5, 5) m/s which is used in Section 4.1 for high maneuverability conditions. The other test conditions are the same as those for high maneuverability conditions.

**Figure 10:**
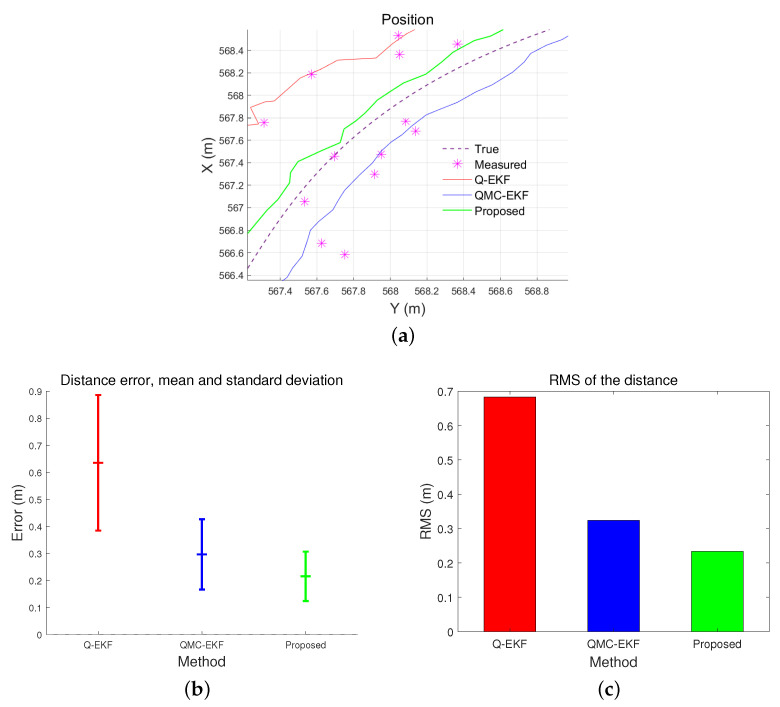
Comparison of position estimation performance under low maneuverability conditions: (**a**) represents the position estimates; (**b**) indicates the mean and standard deviation of the distance error; and (**c**) shows the RMS of the distance error.

Table 4 and Figure 10 compare the trajectory estimation performance by the three methods. They analyze the distance error of the estimated trajectory. The proposed method results in the lowest distance error in average, standard deviation, and RMS, among the three. More importantly, when comparing Figure 5a,b with Figure 10b,c, the performance differences among the three methods are more remarkable under high maneuverability conditions than under low maneuverability conditions. The improvement by the proposed method is more significant when the vehicle moves dynamically.

**Figure 11:**
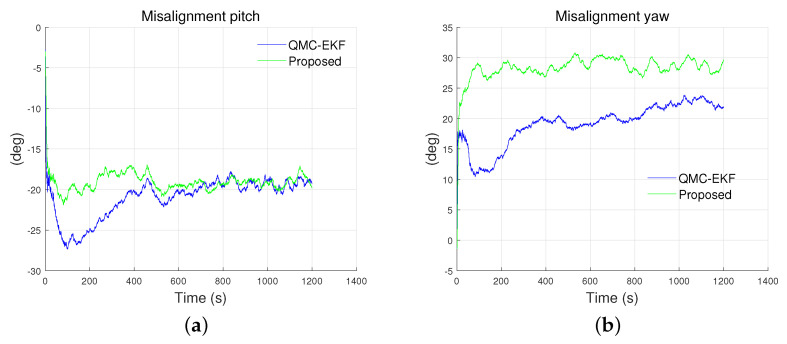
Misalignment estimated under the low maneuverability conditions: (**a**) pitch; (**b**) yaw. True misalignment for the pitch and yaw are −20° and 30°, respectively.

Figure 11 illustrates the estimated results for the misalignment in the pitch and yaw. The proposed method provides a better estimation of misalignment than the QMC-EKF. Specifically, the misalignment in pitch is estimated as −19.0503° and −20.9881° by the proposed method and the QMC-EKF respectively. As for the misalignment in yaw, the estimations are 28.2626° and 19.0502° by the proposed method and the QMC-EKF respectively. Furthermore, the error by the proposed method has less standard deviation and RMS than the QMC-EKF. The RMS of the error in yaw misalignment estimation is 2.2311° and 11.4136° by the proposed method and QMC-EKF respectively. Overall, the proposed method exhibits improved estimation for the estimation of misalignment.

## 5. Experiments and Results

### 5.1. Test Conditions

**Figure 12:**
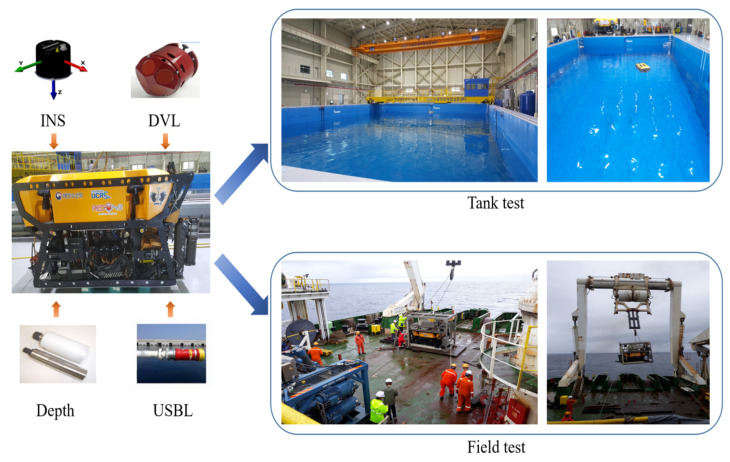
ROV, sensors, and environment for underwater experiments.

The proposed method is tested using a light work-class remotely operated vehicle (ROV). There are two experiments in different environments. The first one is the experiment in a test tank and the second one is the experiment offshore in the East Sea, Republic of Korea. The first one will be called the tank test, and the second one will be called the field test. The detailed specifications of the ROV are listed in Table 5. Figure 12 shows the ROV, sensors, test tank experiment, and the offshore experiment. The ROV is equipped with an INS, DVL, depth sensor, and USBL transceiver and is controlled remotely via human operators. The manufacturers and models of each sensor are listed in Table 6.

In the tank test, the ROV was submerged to a depth of approximately 2.5 m in a water tank and traveled 140 m with a square path. In the field test, the ROV was submerged to the depth of approximately 134 m and operated to travel 200 m with a straight line path.

The position data from USBL is given relative to the USBL coordinate system not a fixed reference coordinate system. Position data provided by USBL is converted to absolute position data using the location and attitude data of the USBL. In the tank test, the USBL is fixed on the construction of the test pool, thus conversion is not required. In the offshore field test, the USBL transceiver is mounted on the ship, necessitating the conversion process. The conversion uses the attitude data from the INS embedded in the USBL system and location data from a DGPS on the ship.

### 5.2. Position Estimation Results

Position estimation results are compared and analyzed. The reference position trajectory is determined based on the USBL data. The position data from the USBL is first interpolated, then the interpolated data is smoothed, and finally, the smoothed data is used as the reference data. The USBL provides location data at a slower rate than the other data from DVL and IMU. Therefore, the position data are interpolated to be synchronized with the other measurement data. Then, to reduce the measurement noise, the interpolated data are smoothed using weighted mean filtering.

**Figure 13:**
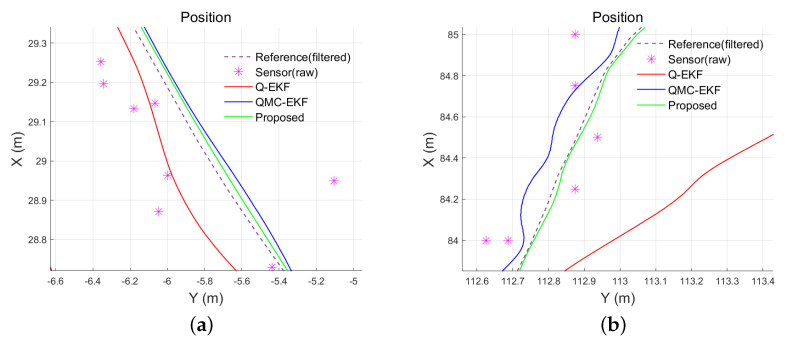
Trajectories estimated using the methods: (**a**) is tank test results for position estimation in xy plane; and (**b**) is field test results for position estimation in xy plane.

Figure 13 shows part of the position estimation result in the tank test and the field test. The line referenced by “Reference(filtered)” indicates the reference trajectory calculated by the method explained above. Figure 13 depicts five sets of data: raw sensor data, filtered reference data, and three trajectories estimated by Q-EKF, QMC-EKF, and the proposed method.

**Figure 14:**
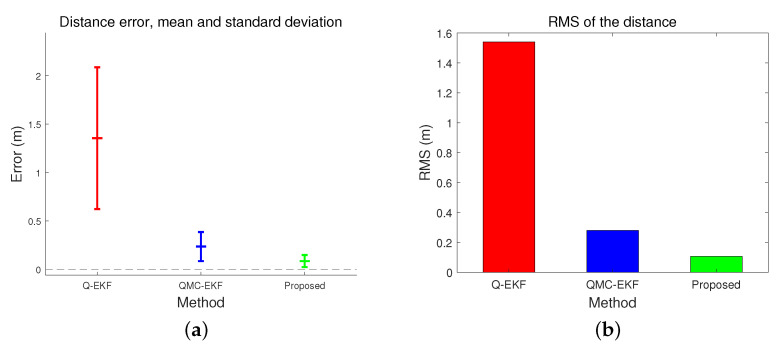
Comparison of distance error in tank test: (**a**) represents the mean and standard deviation of the distance error; and (**b**) indicates the RMS of the distance error.

**Figure 15:**
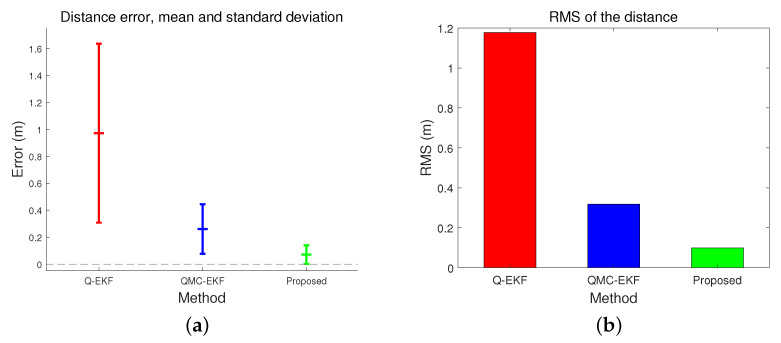
Comparison of distance error in field test: (**a**) represents the mean and standard deviation of the distance error; and (**b**) indicates the RMS of the distance error.

Table 7 compares the distance errors of the estimated trajectories. Figure 14 and Figure 15 depict the analysis shown in Table 7. These results align with the simulation test results, specifically those obtained under conditions of low maneuverability.

### 5.3. Misalignment Estimation Results

**Figure 16:**
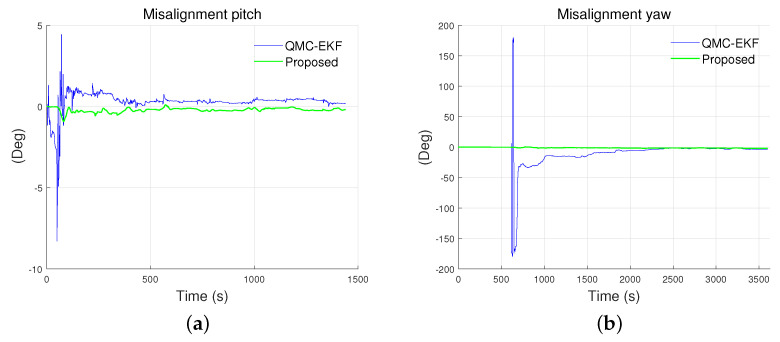
Misalignment estimated using the methods: (**a**) is tank test results for misalignment estimation of pitch; and (**b**) is field test results for misalignment estimation of yaw.

Figure 16 depicts the estimated results for the misalignment of the pitch in the tank test and yaw in the field test. Two plots are presented because only two of the methods estimate the misalignment. Among the methods considered, only QMC-EKF and the proposed method estimate the misalignment, resulting in two available plots. As indicated by ’NA’ in Table 3, Q-EKF does not estimate the misalignment.

As shown in Figure 16a, QMC-EKF exhibits unstable estimates between 46–87 s, during which the ROV started maneuvering. Also, as shown in Figure 16b, QMC-EKF exhibits unstable estimates between 614–701 s, during which the ROV changes its operation mode from dive to horizontal maneuvering. On the contrary, the proposed method improves the estimation of misalignment and provides stable misalignment value. The proposed method estimates the misalignment in pitch as −0.166°, whereas QMC-EKF estimates it as 0.194° which is read after it is stabilized. As for the misalignment in yaw, it is estimated as −1.707° and −2.625° by the proposed method and QMC-EKF respectively. As with the pitch misalignment, it is read after it is stabilized in the case of QMC-EKF. Because the rotation between the INS and DVL does not change during the operation, the misalignment is expected to be kept constant during the operation. The misalignment estimated by the proposed method is kept constant as expected. However, the misalignment estimated by QMC-EKF exhibits an unstable change, attributed to the approximations and adjustments made against the nonlinear nature of the misalignment.

### 5.4. Improvements in Internal Properties

**Figure 17:**
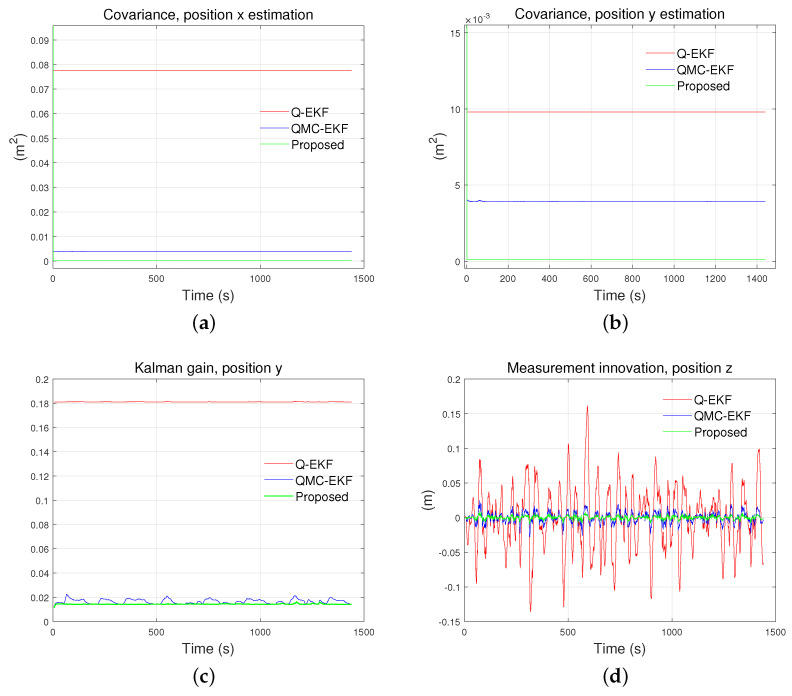
Comparison of internal parameters in tank test: (**a**) is covariance for position x estimation; (**b**) is covariance for position y estimation; (**c**) is Kalman gain for position y; and (**d**) is measurement innovation for position z.

**Figure 18:**
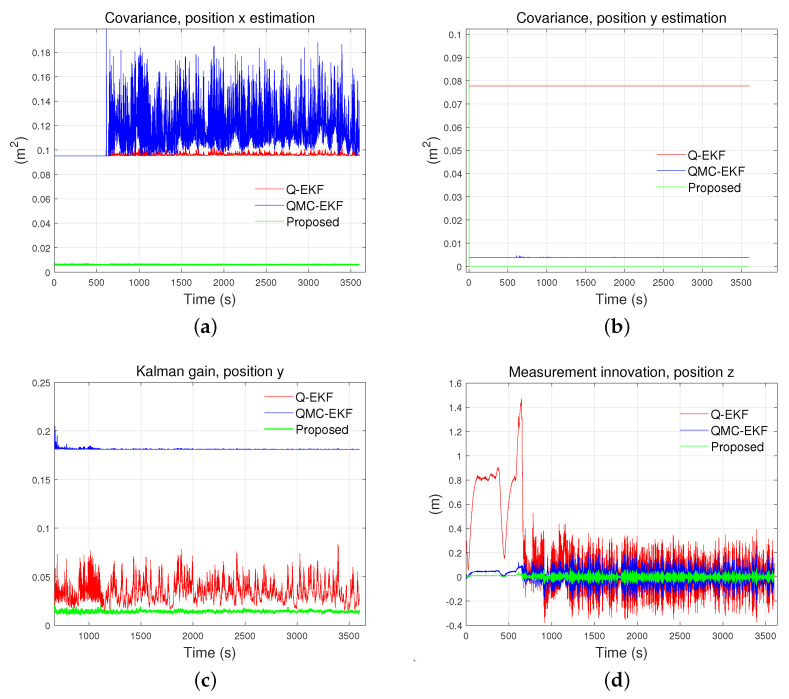
Comparison of internal parameters in field test: (**a**) is covariance for position x estimation; (**b**) is covariance for position y estimation; (**c**) is Kalman gain for position y; and (**d**) is measurement innovation for position z.

The improvement in internal properties is also demonstrated clearly in the experiments. Figure 17 and Figure 18 show the covariance, Kalman gain, and measurement innovation of the estimated position in the tank test and field test, respectively. Covariance, Kalman gain, and measurement innovation are internal parameters specific to the three methods: Q-EKF, QMC-EKF, and the proposed method. These parameters do not apply to the raw sensor data or the filtered reference data, which are included in Figure 13. Consequently, Figure 17 and Figure 18 have only three plots.

In both experiments, the covariance of the estimated x-directional coordinate is the smallest for the proposed method. Also, it is kept steady all the time. The smaller value of covariance indicates that the estimated variable has lower uncertainty. Steady covariance indicates that the estimation is robust to disturbances. Figure 18d shows that the proposed method has superior convergence characteristics, both during the ROV’s diving phase (0–670 s) and its subsequent horizontal motion (after 670 s). As a whole, the results of the experiments show that the stability of the proposed method is superior to the others, while the estimation accuracy is at least comparable to the others.

## 6. Conclusions

This paper presented a Lie theory approach for the navigation of unmanned underwater vehicles that compensates for the misalignment between the INS and DVL. Specifically, we employed the Lie theory for the implementation of the KF and formulated the process and measurement models in Lie groups. The pose and misalignment increments were evaluated and corrected using Lie algebra and exponential operations, and the covariances of the pose and misalignment were appropriately represented in Lie algebra. Consequently, the proposed method enhances the estimation performance in both the estimation accuracy and internal properties. Specifically, the use of Lie theory brings about improved convergence of the covariance, Kalman gain, and measurement innovation. The improvements were validated through simulation and field experiments.

In future research, we plan to apply Lie theory to tracking surface vessels. Applying Lie theory to the tracking of surface vessels is crucial, particularly when precise tracking is required during high-intensity maneuvers. This is because, in the absence of Lie theory, the highly dynamic process amplifies approximation errors, leading to the breakdown of the estimation procedure’s stability. It is expected that the use of Lie theory will provide remarkable improvement in this case.

## Figures and Tables

**Table 1:** Analysis of the position estimation error in the NED coordinate frame (m).

Stat	Di.		Position	
		Q-EKF	QMC-EKF	Proposed
Mn	x	−0.9521	−0.0161	−0.0109
	y	−0.9562	−0.0183	−0.0117
	z	−1.0157	−0.0238	0.0007
Std	x	1.1995	0.4284	0.2266
	y	1.1828	0.4272	0.2258
	z	1.0719	0.4443	0.2322
RMS	x	1.5314	0.4287	0.2269
	y	1.5209	0.4276	0.2261
	z	1.4766	0.4450	0.2322

**Stat: Statistic. Di.**: Direction. Mn: Mean of the error. Std: Standard deviation of the error.

**Table 2:** Comparison of distance error under high maneuverability conditions (m).

Stat	Distance		
	Q-EKF	QMC-EKF	Proposed
Mn	2.6026	0.6915	0.3617
Std	0.2558	0.2941	0.1602
RMS	2.6151	0.7514	0.3956

**Table 3:** Analysis of the error in estimated attitude and misalignment (degrees).

Stat	Di.	Attitude			Misalignment		
		Q-EKF	QMC-EKF	Proposed	Q-EKF	QMC-EKF	Proposed
Mn	ϕ	−0.2220	−0.0379	0.0018	NA	133.8953	−4.9228
	θ	−1.3084	−0.0180	−0.0064	NA	−4.9177	0.1686
	ψ	1.6601	0.0062	−0.0030	NA	10.9918	−0.4202
Std	ϕ	1.7398	1.6499	0.4997	NA	87.1410	10.5798
	θ	1.9603	1.2131	0.6366	NA	9.0491	0.7762
	ψ	2.0898	1.2046	0.6383	NA	11.8913	1.0958
RMS	ϕ	1.7539	1.6503	0.4997	NA	159.7545	11.6690
	θ	2.3569	1.2132	0.6366	NA	10.2990	0.7643
	ψ	2.6689	1.2046	0.6383	NA	16.1933	1.1736

ϕ: Roll, θ: Pitch, ψ: Yaw. NA: Not Applicable.

**Table 4:** Comparison of distance error under low maneuverability conditions (m).

Stat	Distance		
	Q-EKF	QMC-EKF	Proposed
Mn	0.6351	0.2964	0.2153
Std	0.2508	0.1301	0.0914
RMS	0.6828	0.3237	0.2339

**Table 5:** ROV specifications.

ROV Specificaitions	
Dimension	(L) 2000 mm (W) 1300 mm (H) 1500 mm
Weight	1500 kg
Depth rating	2500 m
DOF	6 (Surge, Sway, Heave, Roll, Pitch, Yaw)
Speed	Forward: 2.5 knots, Lateral/Heave: 2 knots

**Table 6:** Sensor manufacturers, models, and specifications.

Sensor	Manufacturer and Model	Specifications
INS	ADVANCED NAVIGATION	Roll & Pitch Accuracy 0.01°
	Spatial FOG	Heading accuracy 0.01°
DVL	Teledyne RD Instruments	Velocity range 10 m/s
	Navigator Doppler Velocity Log	Velocity resolution 0.1 cm/s
Depth	Digiquartz technology, Submersible	0.01% full scale accuracy
	Depth Sensors Series 8000	$1\times 10^{-8}$ Resolution
USBL	Sonardyne, RANGER 2 MF	Range accuracy 2.5% slant range
	GYRO USBL 5000/WSM6+	Position repeatability 0.1% slant range
	Type 8370	Relative position accuracy 5cm

**Table 7:** Analysis of the distance error (m).

Stat	Tank Test			Field Test		
	Q-EKF	QMC-EKF	Proposed	Q-EKF	QMC-EKF	Proposed
Mn	1.3553	0.2365	0.0863	0.9718	0.2606	0.0708
Std	0.7311	0.1507	0.0623	0.6637	0.1836	0.0694
RMS	1.5399	0.2804	0.1065	1.1769	0.3187	0.0991

## Data Availability

The raw data supporting the conclusions of this article will be made available by the authors on request.
